# Educational and gender disparities in work, retirement, and disability-pension expectancies: a cross-country analysis of Italy and Finland

**DOI:** 10.1093/geronb/gbag064

**Published:** 2026-04-08

**Authors:** Margherita Moretti, Elena Fabrizi, Angelo Lorenti, Liina Junna, Pekka Martikainen

**Affiliations:** Helsinki Institute for Demography and Population Health, University of Helsinki, Helsinki, Finland & Max Planck—University of Helsinki Center for Social Inequalities in Population Health, Helsinki, Finland; Dondena Centre for Research on Social Dynamics and Public Policy, Bocconi University, Milan, Italy; Helsinki Institute for Demography and Population Health, University of Helsinki, Helsinki, Finland & Max Planck—University of Helsinki Center for Social Inequalities in Population Health, Helsinki, Finland; Department of Political Sciences, University of Teramo, Teramo, Italy; Helsinki Institute for Demography and Population Health, University of Helsinki, Helsinki, Finland & Max Planck—University of Helsinki Center for Social Inequalities in Population Health, Helsinki, Finland; Max Planck Institute for Demographic Research, Rostock, Germany; Helsinki Institute for Demography and Population Health, University of Helsinki, Helsinki, Finland & Max Planck—University of Helsinki Center for Social Inequalities in Population Health, Helsinki, Finland; Helsinki Institute for Demography and Population Health, University of Helsinki, Helsinki, Finland & Max Planck—University of Helsinki Center for Social Inequalities in Population Health, Helsinki, Finland; Max Planck Institute for Demographic Research, Rostock, Germany; (Social Sciences Section)

**Keywords:** Labor market transitions, Retirement timing, Inequalities, Multistate life tables

## Abstract

**Objectives:**

We examined how gender and education shape the distribution of life years spent in full-year or mid-to-low intensity employment, unemployment-inactivity, disability pension, and retirement in two countries with distinct welfare regimes, labor markets, and pension structures.

**Methods:**

Using Finnish population registers and the EU Statistics on Income and Living Conditions (EU-SILC) data linked to administrative records for Italy, we followed individuals from 2005 to 2018 and applied discrete-time incidence-based multistate models. Through the estimated transition probabilities, we derived life expectancy at age 30 and years spent in different labor market states.

**Results:**

Time spent in full-year employment in general was similar across both countries, but with larger differences by education in Finland and a larger gender gap in Italy. Italy experiences a prolonged time in unemployment or inactivity, particularly among women. Italy also has longer disability pension durations, while Finland has more years in retirement, but with substantial gender and educational differences.

**Discussion:**

We advance existing knowledge by providing more granularity on years spent in employment and retirement and using multistate models on large-scale data. We revealed important gender and socioeconomic inequalities in labor market participation and retirement experiences. We also showed important cross-country differences, likely shaped by welfare regimes and labor market structures. These findings highlight the need for targeted policies addressing structural barriers: in Italy, to reduce gender gaps and fragmented careers, also limiting pension adequacy; in Finland, to address persistent educational inequalities in employment and retirement.

Many countries are experiencing rapid population aging, which raises growing concerns about the long-term sustainability of their welfare and pension systems. In this study, we estimate working life expectancy (WLE) for Italy and Finland over the period 2005–2018. WLE is a summary indicator of the number of years an individual can expect to spend in employment, derived from the demographic measure of life expectancy (LE). This approach allows us to capture the length of working life relative to total LE, making cross-country comparisons meaningful. Crucially, this life-course perspective reflects the overall contribution to the sustainability of pension systems. The latter cannot be assessed by only considering retirement age (being the target of many pension reforms aiming at raising the exit age) and without accounting for the full employment history, because it overlooks how much time people actually spend in employment over their lives.

Italy and Finland represent two distinct welfare models (Esping-Andersen, 1990). Italy is characterized by a Southern European familistic welfare model, in which the family, and particularly women, carry out (family) care responsibilities for which the state does not provide adequate support, while Finland is characterized by a Nordic welfare system that is more gender-egalitarian, where public support promotes balance across gender roles in many life domains, including labor market participation. Despite these structural differences, both countries face the consequences of intense population aging, driven by high LE and declining fertility rates; indeed, both Italy and Finland are now among the lowest-low fertility countries (Billari & Kohler, 2004; Hellstrand et al., 2020). Accordingly, both have undergone a series of reforms aimed at ensuring the long-term sustainability of their pension systems (OECD, 2023). These shared demographic trends, combined with distinct pension reforms and differing labor market structures, make the two countries a compelling comparative case study. Examining how structural differences interact with similar demographic pressures can clarify the mechanisms by which welfare systems shape outcomes and thereby inform potential areas of policy intervention.

We examine how gender and education shape the distribution of working and non-working life years in Finland and Italy between 2005 and 2018. Unlike previous studies, we are the first to estimate WLE disaggregated by full-time and mid- to low-intensity employment. This distinction is important for understanding a key aspect of recent labor market developments. In many countries, employment levels have risen in recent years. However, this positive trend is likely driven (in countries such as Italy) in large part by an increase in precarious employment, which often serves as a selective arrangement for disadvantaged groups, such as women and individuals with low educational attainment (Cuccu et al., 2024). This selective expansion may widen socioeconomic gaps and yield limited gains for pension sustainability, given the reduced volume of contributions flowing into the system. To better understand how non-work years are distributed, we estimate and distinguish expected years spent on retirement and disability pensions, as well as being either unemployed or inactive. This detailed overview provides a picture of how employment and retirement years are distributed across life in these two countries and across different population subgroups, therefore providing a complete overview of how demographics and labor market conditions intersect to put pressure on the corresponding pension systems.

## Background

### Working life expectancy

Working life duration and timing of labor market exit for retirement are influenced by different factors (Kohli et al., 1991). Among those, health status, employment opportunities, the social security benefits (such as early retirement options), and individual socioeconomic status (e.g., education, wealth), may influence the choices and constraints an individual faces when considering whether to remain in the labor force or exit through retirement when older (Leinonen et al., 2018; Solovieva et al., 2024). As a result, WLE and the time spent in retirement vary significantly across population subgroups (Dudel & Myrskylä, 2017; Lorenti et al., 2019). Men generally have longer working lives than women; working life expectancies are also longer for individuals with higher socioeconomic status, a gap that is persistent even in the later stages of working life (Leinonen et al., 2018; Nurminen, 2012). Compared to individuals with higher levels of education, individuals with lower educational levels experience more prolonged periods of unemployment or periods outside the labor force throughout their working-age years (Nurminen, 2012). They also face higher risks of labor market exiting, or going in and out of the labor market, for reasons such as unemployment or having poorer health (Schuring et al., 2013). Moreover, they experience higher mortality rates and greater variability in age at death (van Raalte et al., 2011), which reduces the average duration in both employment and retirement. Socioeconomic differences in mortality persist until old age (Hoffmann, 2008) and socioeconomic groups exhibit considerable disparities in their remaining (healthy) life expectancies around the age of statutory retirement (Cambois et al., 2011; Majer et al., 2011; Shkolnikov et al., 2008). Similarly, disparities occur in the number of years spent in retirement because of the differences in the time at which individuals can actually retire and at which they exit through death. The duration of working life is inherently connected to the timing and length of retirement or disability retirement, which reflect and reinforce the same socioeconomic and health inequalities observed during working life (Schuring et al., 2013; Wahrendorf et al., 2013). Therefore, examining working life duration alongside retirement, disability pensions, and unemployment or inactivity is essential to fully capture the dynamics of labor market participation, exit, and the duration of each stage.

Previous studies (such as Leinonen et al., 2018; Junna et al., 2024; Lorenti et al., 2019) have documented gender and educational inequalities in WLE in Finland and Italy (separately). However, these studies typically rely on aggregated measures (such as prevalence-based approaches) or do not account for a detailed range of labor market states. Our study is the first to offer a comparative analysis of these two countries using individual-level administrative data (using whole-population data for Finland), while employing detailed measures of employment intensity and multistate modeling.

### Context of the study

Italy and Finland both face challenges related to population aging, and in both countries, health and LE are marked by socioeconomic inequalities. In Italy, LE is among the highest in Europe (Eurostat, 2022), with women and men having a LE of ∼85 and 80 years, respectively, in 2021 (Human Mortality Database, 2025). However, there are socioeconomic disparities in survival, for example, individuals with lower levels of education generally live ∼1.5 to 3 years less than their more educated counterparts (Caselli et al., 2021; Lallo & Raitano, 2018; Luy et al., 2011), also associated with a smaller proportion of life spent in good health (Moretti & Strozza, 2022). In Finland, LE at birth is also high, with women and men living ∼84 and ∼79 years, respectively, in 2021 (Human Mortality Database, 2025), but with even larger disparities by socioeconomic status. As an example, the LE gap between the extreme income quintiles in 2018–2020 was around 11 years for men, and 6 years for women (Tarkiainen et al., 2024), and in 2020, the gap between those with tertiary education and those without secondary education was around 7 years for men and 5 for women (Junna et al., 2024).

### Description of the labor market and pension systems in the two different countries

#### Italy

**Figure 1:**
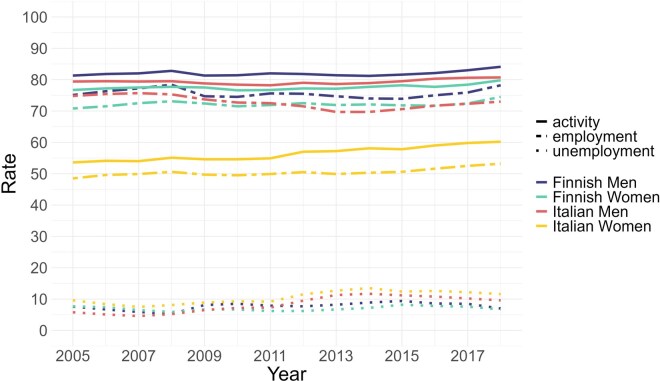
Employment, unemployment, and activity rates in Finland and Italy by gender, 2005–2018. *Note*. In our expectancy analysis, unemployment and inactivity are aggregated due to data constraints in the Italian dataset; however, the Eurostat indicators shown here follow harmonized international definitions with different denominators and therefore cannot be combined in the same way. In this case, as in the Eurostat definitions, “The active population (labour force) is defined as the sum of employed and unemployed persons. The activity rate is the percentage of active persons in relation to the total population”; “The employment rate is the percentage of employed persons in relation to the total population”; “The unemployment rate is the number of unemployed persons as a percentage of the active population.” *Source*: Eurostat website (https://ec.europa.eu/eurostat/databrowser/bookmark/e34e5649-6267-4136-9958-abddbd954052? lang=en&createdAt=2026-02-17T13:19:15Z and https://ec.europa.eu/eurostat/databrowser/bookmark/d5b0a66d-17fc-41ae-b903-79f6888cc96b? lang=en&createdAt=2026-02-17T13:20:17Z).

Over the past few decades, the Italian labor market has experienced important transformations, including a rise in (but still low) female labor force participation, delays in entering the workforce due to extended periods of education, increased labor market flexibility, and noticeable shifts toward more fragmented employment patterns and less stable careers. However, large gender differences in labor market participation remain, and an increasing share of people in precarious employment (especially young workers) frequently lack adequate social protection in case of unemployment or disability, reflecting a strong social protection dualism (Barbieri, 2011). The consequences of such disparities in social protection were particularly evident during the Great Recession. Indeed, the financial crisis of 2007–2008 had a severe impact, leading to increased unemployment and economic insecurity, especially among young people and women (Lorenti et al., 2019). Prolonged periods of unemployment and a greater likelihood of precarious employment have an impact on the subsequent work trajectories and retirement prospects, as a consequence of reduced contributions. Opportunities for stable, long-term employment in general are limited, especially for women and younger workers (Fabrizi & Rocca, 2024). Italian women, who have much lower labor force participation rates than men, are also likely to be employed in part-time or temporary jobs (Boeri et al., 2005), which offer less security and lower pensions, and this is partly explained by interruptions in their careers for caregiving responsibilities (Mussida & Patimo, 2023). Caregiving responsibilities for women have scarring effects that last until retirement and increase with the number of children (Lorenti et al., 2024). As reported in the Ageing Report 2024 (European Commission: Directorate General for Economic and Financial Affairs [DG ECFIN], 2023), the labor force participation rate in the age class 20–64 is only ∼60.5 for women in 2022 (compared to 80.4 for men). This level is lower than the European average (DG ECFIN, 2023), and the differential between the two genders in their labor force participation rates was the second highest among EU countries in 2020 (see also Figure 1).

The Italian pension system has undergone several reforms (from 1992 to 2011) that produced a radical transformation, modifying most of the basic parameters for retirement, with the aim of reducing the pressure of an aging population on public finances. According to these reforms, the system gradually moved from a Defined Benefit to a Notional Defined Contribution rule (Mazzaferro, 2023). Notably, the majority of the pension system reforms marked a significant shift in raising the statutory retirement age, tightening the eligibility requirements to retire. The observed average labor market exit age did not increase much overall in the last few years, and in 2022, it corresponded to around age 64 for both genders, only slightly higher than in previous years (DG ECFIN, 2023).

#### Finland

The Finnish labor market is considered relatively flexible, but by European standards, there are still strong regulations that protect employees. However, there is some indication of a shift from an egalitarian welfare regime toward a policy that emphasizes activating those outside paid employment. Following the deep recession of the mid 1990’s, Finland has, for example, tightened the eligibility criteria for basic unemployment insurance (Kananen, 2012). Also, the share of those in temporary employment is considered relatively high, especially among younger workers (Eichhorst et al., 2017). While these shares have increased over time, during our study periods, no major reforms were introduced to reduce temporary employment (Eichhorst et al., 2017).

The gender gap in labor market participation in Finland has long been among the smallest in Europe: in 2018, the employment rate among men aged 20–60 was 77.2% compared to 73.4% among women, a gap of less than four percentage points (Eurostat, 2024; Figure 1). The high female participation rate, especially in full-time employment, is often attributed to parental leave and public daycare systems, which have allowed women flexibility to combine work with childcare (Kyyrä & Pesola, 2020). On the other hand, the participation rates among Finnish women with young children are still low compared to the other Nordic countries (Kyyrä & Pesola, 2020), possibly because of the limited availability of part-time jobs. Moreover, compared to the other Nordic countries and even the OECD average, the Finnish unemployment and long-term unemployment rates have been consistently high during the 2000’s (OECD, 2019, 2021), varying between 6% and 10%. After being hit hard by the 2008 financial crisis (OECD, 2019), the Finnish economy has stagnated, resulting in the growth of structural unemployment (Kyyrä & Pesola, 2020).

During the 2000s, several pension reforms were introduced, but the fundamentals of the Finnish pension system have remained unchanged. The general retirement age became flexible between ages 63 and 68, and the options for early retirement were reduced. Alternatively, individuals can access disability pension (in case of diagnosed diminished working capacity) and special pensions for farmers (Kuivalainen & Kuitto, 2022) at earlier ages. The result of recent reforms was an increase in the average age at retirement and in labor market participation rates among those aged 50 and older (Kuivalainen & Kuitto, 2022; Kyyrä, 2015; Kyyrä & Pesola, 2020). Conversely, labor market participation has not increased at a similar pace among younger age groups (Eurostat, 2024; Junna et al., 2024; Kyyrä & Pesola, 2020).

To aid the interpretation of the results, Table  1 presents a summary of the institutional, labor‑market, and pension characteristics of Italy and Finland. Figure  1 displays the activity and employment rates for both countries using Eurostat data.

**Table 1:** Summary of key institutional, labor market, pension system, and demographic characteristics of the study samples.

Dimension	Italy	Finland
Welfare regime (Esping-Andersen, 1990)	Mediterranean, family-oriented welfare state	Nordic universalistic welfare regime
Female employment rate (20%–64%, in 2005–2018)	Low, around 50%	High, around 70%
Gender gap in employment	Persistently large	Relatively small
Unemployment and inactivity patterns	High and persistent inactivity, especially among women	Lower inactivity; structural unemployment relatively high, but lower than in Italy
Labor market structure	Segmented, precarious, and intermittent employment; strong social protection dualism	Relatively flexible but still strong regulations; relatively high temporary employment, especially among young people
Pension system structure	Shift from Defined Benefit to Notional Defined Contribution; increasing statutory retirement age; aims to tighten eligibility	Flexible retirement age (63–68); increasing statutory retirement age; reduced early retirement pathways
Life expectancy and socioeconomic inequalities in mortality	High; quite moderate socioeconomic inequalities in mortality	High; strong socioeconomic inequalities in mortality
Demographic context	Very low fertility; rapid population aging	Very low fertility; rapid population aging

*Note*. See Figure 1.

### Objectives

In this study we aimed to: (i) quantify how life expectancy from age 30 onward is distributed across five states (a) working full-year, (b) working mid-to-low intensity, (c) unemployment and inactivity, (e) disability pension, and (f) retirement, and compare these patterns between Finland and Italy; (ii) investigate how time spent in each of these states varies by gender and educational attainment and their intersection, in the two countries.

## Data and methods

### Data

For the reference populations, we selected Finnish and Italian adults aged 30 and older in 2005. For Italy, the original representative sample of the whole population comes from the 2005 Italian Survey on Income and Living Conditions (IT-SILC) (∼47,000 individuals aged 30+ in 2005). This dataset covers the population living in households (non-institutionalized), and the total response rate in 2005 is almost 85% (https://gesis.org/en/missy/materials/EU-SILC/documents/quality-reports). These individuals were then linked with administrative registers of the National Institute for Social Security (INPS) to track work history information and mortality—that is, only individuals with contribution histories of unemployment, employment, and/or pensions were linked (93% of the original sample). After exclusions, the sample was around 40,000 individuals. For Finland, we used data for the entire population from Finnish population registers (more than 3 million adults aged 30+ in 2005). For both countries, we followed each individual from 2005 to 2018. We censored individuals upon outmigration and included individuals returning to the study population during our observation timeframe. The observation window was determined primarily by data availability for Italy, as the data were only available up to 2018. Moreover, as the Italian data do not allow us to distinguish between inactive and unemployed individuals, we collapsed the two categories.

We used the labor force status during each calendar year as our outcome variable. At each age and year, individuals were classified among five mutually exclusive states: (1) work full-year (employed for more than 350 days in the year); (2) work mid to low intensity (employed between 30 and 350 days in the year); (3) unemployed, inactive, or work less than 30 days in the year; (4) in disability pension; and (5) retired (old-age pension). Those who die at any time in the year were classified as deceased. Supplementary Material includes further explanations of the states (see Supplementary Methods 1).

Educational level is defined by the highest degree obtained and classified in three categories, “low,” “mid,” and “high,” which correspond to International Standard Classification of Education-2011 (ISCED) 0–2, ISCED 3–4, and ISCED 5–8, respectively.

Table 2 describes the populations analyzed in this study, including the prevalence of working states by age classes, country, and gender.

**Table 2:** Prevalence (%) in the different states by age classes, for Finnish and Italian men and women.

Group	Age classes	Full year	Mid to low intensity	Unemployed or inactive	Retired	Disability pension	Total
Finnish men	30–49	74.5	12.6	8.5	0.0	4.5	100.0
	50–64	54.4	8.2	13.2	6.0	18.3	100.0
	65+	0.2	0.0	0.7	98.9	0.1	100.0
Italian men	30–49	70.3	7.1	17.0	0.8	4.9	100.0
	50–64	21.3	1.9	10.6	56.0	10.2	100.0
	65+	0.3	0.0	5.5	69.4	24.8	100.0
Finnish women	30–49	67.7	17.6	11.1	0.0	3.6	100.0
	50–64	55.1	8.3	13.7	6.7	16.3	100.0
	65+	0.1	0.0	1.5	98.3	0.1	100.0
Italian women	30–49	53.4	7.3	34.8	1.2	3.4	100.0
	50–64	13.1	1.2	30.6	48.4	6.7	100.0
	65+	0.1	0.0	15.0	58.6	26.2	100.0

### Analysis

We estimated period life expectancy and (unconditional) status-specific expectancies through discrete-time incidence-based multistate models, by gender and education. First, we estimated transition probabilities (to describe movement between states) using multinomial logistic regression, stratified by gender. The covariates included the individual’s age, squared and cubic age, and education. Second, we estimated expectancies using the standard (discrete-time) Markov-Chain approach (Dudel, 2021; Kemeny & Snell, 1983). See Supplementary Methods 2 for more details on methods. All analyses were performed in STATA (StataCorp, 2023) using the *dtms* package (Schneider, 2023), and figures were produced in R (R Core Team, 2021).

## Results

### Life expectancy in Italy and Finland

**Figure 2:**
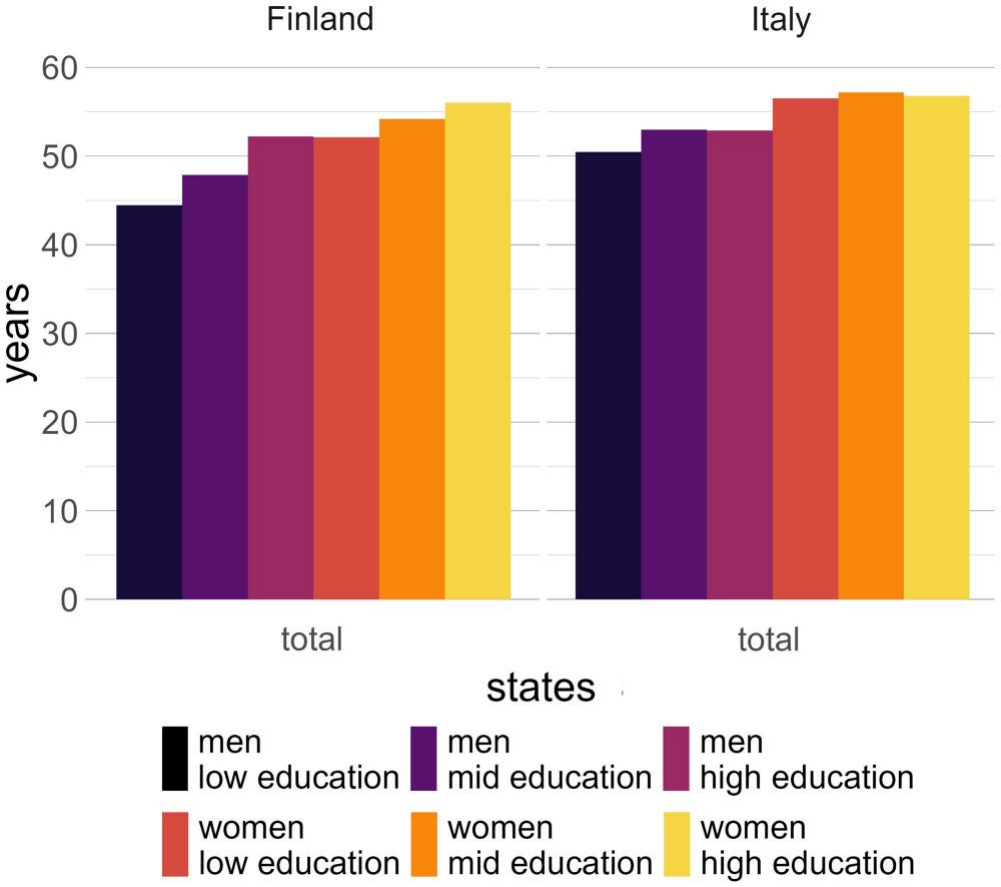
Overall life expectancies at age 30 between 2005 and 2018 for Finnish and Italian men and women by level of education. *Source*: Authors’ own computation.

As shown in Figure 2, Italy has a higher LE than Finland at aged 30, at ∼57 years for women and ∼53 years for men, compared to the corresponding 54 years and 48 years in Finland. Moreover, Italy has lower differentials in LE by level of education: almost null among women and ∼3 years among men when comparing the educational extremes. The inequalities are more prominent for Finnish women and men at ∼4 and ∼8 years, respectively. These differences in overall LE provide an important reference for the subsequent results that partition LE.

### Years spent working (full-year and mid-to-low intensity)

**Figure 3:**
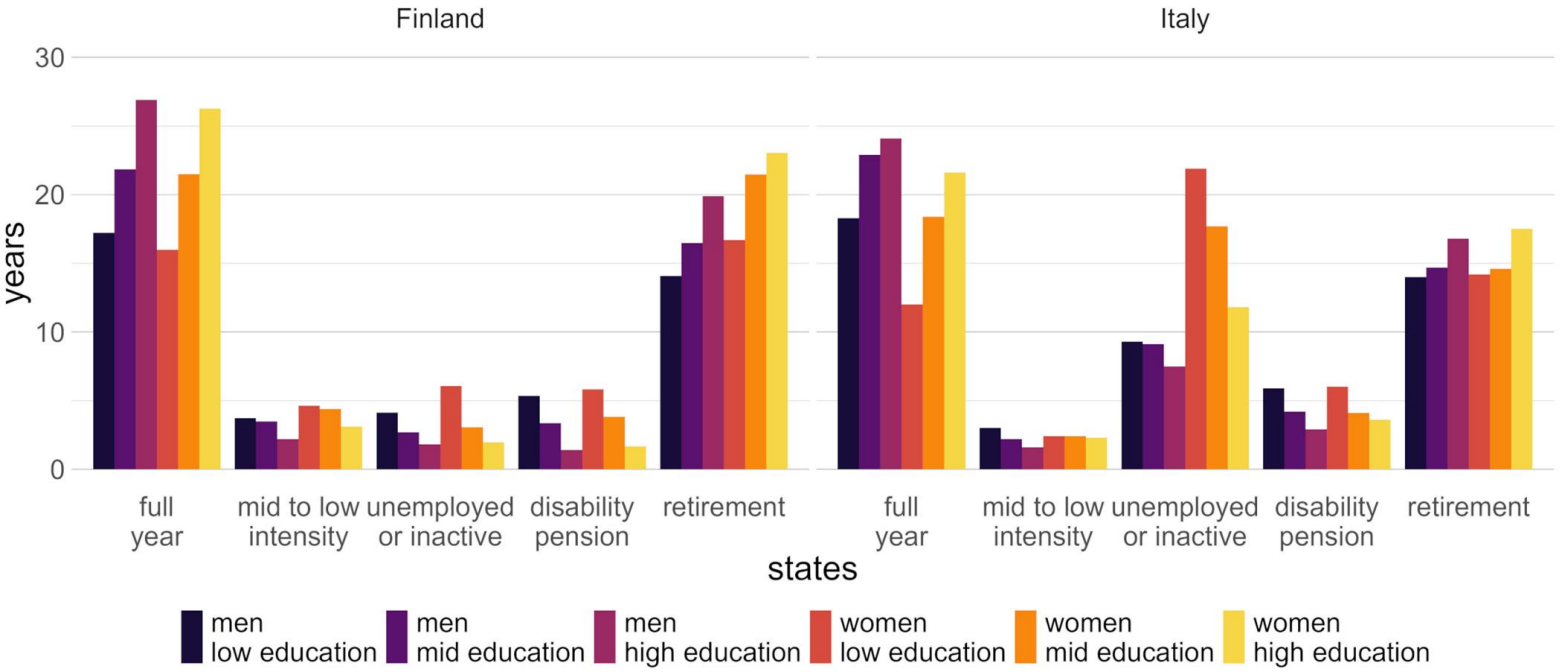
State expectancies at age 30 between 2005 and 2018 for Finnish and Italian men and women by level of education. *Source*: Authors’ own computation.

While Finns have lower LE at age 30, the years spent working full-time were similar to those of their Italian counterparts (Figure 3). In Finland, men and women at aged 30 can expect to experience ∼22 and ∼20 years working on a full-year basis (more than 350 days per year), respectively. In Italy, the corresponding estimates were ∼22 years for men and ∼17 years for women. Thus, Italian women have fewer years in this state than their Finnish counterparts.

The average number of years working full year is higher in Finland than in Italy for highly educated men and all women, irrespective of education. The gap between the educational extremes in full-year work expectancy is stronger in Finland than in Italy for men and more pronounced for women than for men in both countries. The largest educational differential is thus observed for Finnish women, at more than 10 years between the educational extremes. For men, the differentials are ∼6 years in Italy and almost 10 years in Finland. Thus, Finnish men and women have a similar number of years in this state, while, on the contrary, in Italy, men have higher levels compared to women.

The average number of years working low or mid intensity is slightly higher in Finland than in Italy, but still low in both countries (less than 5 and 3 years, respectively). For women, however, the level is almost half in Italy compared to Finland. The values are 1.5 years higher for the low-educated group than the highly educated group for both genders in both countries, except for Italian women, for whom there is almost no difference.

### Years spent in unemployment or inactivity

The most striking difference between the two countries was the average number of years spent in unemployment or inactivity. This is particularly high for Italy and especially for Italian women: 22 years for the low-educated group (compared to more than 56 years of LE) and almost 12 years for the highly educated group (compared to 57 years of LE). Interestingly, low-educated Italian women live almost twice as long in unemployment or inactivity as they do in full-year employment. The opposite was observed for men and highly educated women in Italy: for men, the time spent working full year is two to more than three times higher than the time they spend in unemployment (for low- to highly educated, respectively), while for highly educated women it is about two times longer. For Italian women, in fact, there were large differences by level of education, with the lowest educated spending 10 years longer in this status than the highly educated, whereas for men, the differential was ∼1 year. Compared to Italy, the expected years in unemployment or inactivity for Finnish men and women were much lower, with values ranging for low-educated to highly educated from 4 to almost 2 years for men and from 6 to 2 years for women.

### Years spent in disability pension and retirement

The number of years spent in disability pension is higher in Italy than in Finland for both women and men and across educational levels, except for low-educated women. In Italy, the disability pension expectancy is ∼6 years for those in the low-educated group for both genders, almost 3 years and 4 years among highly educated men and women, respectively. For Finnish men in the low-educated group, the expectancy is ∼5 years, while for the highly educated group is slightly more than 1 year. For Finnish women, the number of years on disability pension is 5.5 and 1.5 years for the low-educated and highly educated groups, respectively. In Italy, the gender difference was thus almost null for low-educated and mid-educated groups, but not negligible for the highly educated group (where women spend almost one year longer in this state). In Finland, the gender gap is similar across education levels (but slightly higher for the low-educated and mid-educated groups), with women spending about half a year more than men. In disability pension, the disadvantage of the low-educated group spending more time in this state was greater in Finland (∼4 years for men and women); for Italy, the corresponding values were ∼3 years for men and ∼2 years for women.

The number of years spent in retirement in Italy was ∼14 years for the low-educated group, ∼15 years for the mid-educated group, and ∼17 years for the highly educated group, for both genders. While gender differences were very small in Italy, they were greater in Finland, with women having ∼3 more years in retirement (low-educated: 17 years for women vs 14 years for men; highly educated: 23 years for men vs 20 years for men), likely due to their longer LE. The differences by level of education, with those who are highly educated spending more years in retirement, were also greater in Finland than in Italy. The differentials are ∼6 years for Finnish women and men, ∼3 years for Italians, and higher for women in both countries. Therefore, in Italy, individuals spend on average more years in joblessness and in disability pensions, while in Finland, they spend on average more years in retirement. State-specific results expressed in relative terms (state-specific number of years over total LE) are provided in Supplementary Table 1 and Supplementary Figure 1 (see online supplementary material), and lead to similar conclusions.

## Discussion

In line with aim (i), we quantified how LE at age 30 is distributed across six labor market and retirement states and compared these patterns between Finland and Italy. Italians have higher overall LE at age 30 than Finns. Both countries show similar overall durations in full-year employment among men; however, Finns (and particularly the highly educated) spend slightly more years in this state than their Italian counterparts. Finns also spend slightly more time in mid-to-low intensity employment, especially women, than Italians. Inactivity and unemployment durations are strikingly longer in Italy, particularly for Italian women. Finally, Italians spend less time than Finns in retirement but more years in disability pension. Related to aim (ii), we find inequalities by gender, education, and their intersection in these estimates. Educational inequalities in full-year employment are pronounced in both Finland and Italy, but are especially large in Finland and among women in both countries. Lower-educated men spend more time in mid-to-low intensity employment than higher-educated men in both Italy and Finland. Gender differences are notably wide in Italy, where women, and particularly those with low education, are most disadvantaged. Intersecting gender and educational disadvantages, low-educated women in Italy spend, in fact, nearly twice as much time in inactivity or unemployment as they do in full-year employment. Although Finland has shorter durations of unemployment and inactivity overall, strong educational disparities also remain in this state and, again, especially among women. Important educational and gender differences are found for both countries, and especially for Finland, also in the years spent in retirement (where men and those with a low education level spend less time in this state).

### Comparison with the literature

Our results are in line with previous findings, including those from Leinonen et al. (2018) and Junna et al. (2024) in Finland and Lorenti et al. (2019) in Italy. Specifically, Leinonen et al. (2018) highlight that, in Finland, women at age 50 spend a greater amount of time in retirement than men (in line with our results), reflecting, among other factors, their longer LE. Men, on the other hand, have shorter LE and spend a larger proportion of their remaining life in work due to higher mortality rates in retirement. Similar to Junna et al. (2024), we found great social inequalities in WLE. In Italy, we observe dynamics similar to those discussed by Lorenti et al. (2019), especially the disadvantage women face in the labor market. These authors, indeed, highlight how Italian women are more likely to be in temporary and flexible employment, with limited transitions to permanent positions (Istat, 2013), which significantly affects their WLE and economic stability and reflects in the striking gender differences we found for the country. This is a result of the persistent gender gaps in activity and employment rates, as described in Table 1 and Figure 1. WLE in Italy is relatively low for young people and women (Lorenti et al., 2019). This pattern is commonly attributed to very low employment rates at younger ages and the likely scarring effect of long-term unemployment (Bell & Blanchflower, 2011; Mroz & Savage, 2006). Care duties play a significant role in explaining the large gender gap, as they contribute to cumulative employment disadvantages for mothers, which increase with the number of children and persist until retirement (Lorenti et al., 2024).

These studies also emphasize how social inequalities in WLE are linked to differences in labor market participation, health challenges, and mortality for both countries. Our study further contributes to this literature by showing the extent to which more disadvantaged groups experience higher rates of unemployment and disability retirement (Junna et al., 2024; Leinonen et al., 2018; Nurminen, 2012; Solovieva et al., 2024).

The observed differences should be interpreted in light of the institutional, labor market, and pension contrasts, as summarized in the introduction and in Table 1 and Figure 1. In terms of the path that has led to these outcomes, the evolution of employment and unemployment rates over the past decades has diverged between Finland and Italy (as shown in Figure 1), with Italy recovering more slowly after the crisis and exhibiting persistently longer times spent in unemployment or inactivity (Eurostat, 2024). While unemployment differences between the two countries are pronounced, particularly for women, the longer time Italians spend in inactivity or unemployment appears to be driven more by persistent gaps in inactivity rates (Eurostat, 2024). These gaps remain striking over time, especially for women, reflecting a persistent group of individuals in Italy who either never enter the labor market or experience unstable and fragmented employment, substantially prolonging their time spent in inactivity or unemployment.

Our study is based on large-scale data; in the Finnish case, it encompasses the full population, offering a highly reliable foundation for our estimates, especially when compared with the more limited data sources typically used in similar research. In the Italian case, we relied on an innovative and unique, but restricted, integrated dataset that links survey and administrative records, enabling a more detailed and accurate reconstruction of individuals’ labor market careers. Building on and extending the work of Lorenti et al. (2019), Junna et al. (2024), and Leinonen et al. (2018), our analysis advances the literature in several important ways. While these studies provide valuable estimates of WLE, they rely on broader classifications of labor market states (e.g., employment vs non-employment) or focus on specific age ranges (e.g., from age 50 onward) and do not distinguish between different employment intensities or separate disability pension from other non-employment states. First, we introduced more fine-grained measures of employment by distinguishing between full-year and mid-to-low intensity employment, based on the number of days in employment per calendar year. This allowed us to capture variation in employment patterns that had previously been overlooked, particularly across gender and educational groups in both countries. Second, the inclusion of disability pension, alongside the employment and retirement states, offered insights into a dimension that is often overlooked. Third, by applying multistate modeling (similarly to Lorenti et al., 2019) instead of the more commonly used prevalence-based approaches, we were able to model dynamic transitions across a wider set of labor market and retirement states. In other words, our study adds new insights by incorporating recent and extensive data, a greater number of states in the analysis, identification of educational differences (similar to previous studies), and more refined methods. These together offer a deeper and more up-to-date perspective on working, unemployment, retirement, and disability pension expectancy, and their gender and social gradients, in two countries with different welfare regimes.

### Implications for policy

Our study emphasizes the importance of addressing gender and socioeconomic differences in policies aimed at ensuring sufficient employment for the sustainability of the welfare systems and promoting fairness so that all individuals can have both adequate employment and retirement years. Alternative measures beyond the commonly proposed increase in the statutory retirement age may also be crucial in achieving this fairness and sustainability. In fact, the gains in LE do not necessarily lead to more time spent economically inactive; they can instead result in an increase in time spent in employment (Leinonen et al., 2018) due to a reduction of mortality at ages before statutory retirement. Overall, our results suggest that reducing these inequalities requires coordinated action across labor market, pension, gender equality and family, education, and health prevention policies and strategies. In the Italian case, structural barriers (Pacifico et al., 2018), such as hindrance into early entry (or to re-entry) into the labor market, reduced employment opportunities, frequently unstable, intermittent, and precarious employment, employee working fewer hours than they would like and limited childcare availability (Istat, 2020) may all contribute to gender and educational divides in employment and in retirement in later life. Policies that strengthen work–family support, such as improving childcare availability and affordability, and promoting more balanced maternity and paternity leave arrangements (as in Finland), may help reduce these structural inequalities. Discontinuous careers (especially among women and individuals with low education levels) can significantly lower pension entitlements or, in some cases, prevent early retirement altogether when minimum pension thresholds are not met. Measures aimed at addressing the above-mentioned barriers and labor market fallacies would help close these gaps and improve the fairness, sustainability, and equity of working life. In the Italian context, increasing women’s participation in the labor market could mitigate their prolonged periods of involuntary unemployment and inactivity, thereby enhancing the system’s sustainability from this perspective. Meanwhile, in Finland, the study shows relevant educational inequalities both in the years spent inside/outside the labor market and the years spent in retirement. Finland, already relatively gender-equalitarian, could instead benefit from targeted efforts to improve employment opportunities for low-educated individuals, for example, through strengthened lifelong learning, expanded active labor market programs for low-educated workers, policies facilitating transitions from temporary to stable employment, and wage subsidies. Finally, research has indeed shown that disability-free life expectancy around retirement age in Italy (and other countries such as France) has not always kept pace with the increase in overall LE at the same ages (Cambois et al., 2011; Moretti et al., 2026). As a result, some workers may reach the retirement eligibility age in poor health, leading to early exit from the labor market through disability pensions. If health outcomes at later working ages do not improve for all, policies aimed at raising the retirement age would likely fail to extend working life and instead could lead to more years spent in disability pension (Serrano-Alarcón et al., 2023), especially for the more disadvantaged. Similar concerns can also apply to Finland and, in general, to other national contexts. Taken together, these findings indicate that extending working life in an equitable way requires broader structural interventions addressing employment stability, access to the labor market, gendered career interruptions, and health at older ages.

### Methodological considerations

In the interpretation of our results, two data and methodological considerations need to be considered. First, regarding the data, the AD-SILC dataset is based on the Italian module of the SILC sample, which means it reflects a sample rather than the entire Italian population (Istat, 2021). While the sample is large and representative of the Italian population, our estimates of total LE are slightly higher than those of the Italian National Institute of Statistics (ISTAT), similar to other works with similar data (Moretti et al., 2026). This is very likely the result of our data excluding the institutionalized population. By contrast, Finnish register data cover the whole population. Second, in terms of methodology, we adopt the Markovian assumption, where the probability of transitioning to a given state depends only on the current age and state. While this assumption has limitations, previous applications have demonstrated that unconditional expectancies derived from this approach still provide consistent estimates even when the underlying data-generating process is non-Markovian (Shen & O’Donnell, 2024). Furthermore, compared to studies that use the prevalence-based Sullivan method (Imai & Soneji, 2007; Sullivan, 1971), our approach makes fewer assumptions, leading to more accurate and robust results.

### Future studies

This work opens avenues for future studies to explore the complex relationships between health, gender, education, and labor market participation. Health status plays a crucial role in an individual’s ability to fully participate in the labor market, and its influence is particularly relevant in disparities by level of education. We showed that there are important educational and gender differences in the length of life and in the disability pension expectancies. Future studies should, indeed, delve deeper and jointly consider health and labor market participation to understand how health disparities contribute to differences in employment opportunities and durations. Moreover, future works could give new insights into gender differences in labor market participation by considering full-time versus part-time, precarious, and other types of employment. This would be especially important for countries like Italy, where structural barriers to women’s full participation in the labor market limit employment opportunities. As more women from recent cohorts are entering the labor market, it is also crucial to assess whether new barriers or obstacles persist and how they evolve. Furthermore, given the heterogeneity of population subgroups also defined by education and occupation, future research should explore how shifts in occupation and technological advances, such as automation, influence gender and socioeconomic disparities in workforce participation. This will help identify how these factors may impact various population subgroups, and whether they exacerbate or mitigate existing inequalities.

## Supplementary Material

gbag064_Supplementary_Data

## Data Availability

The data used in this study are not publicly available due to data protection regulations in Finland and Italy. Access to Finnish administrative data can be requested through Statistics Finland, and access to Italian data through the Department of Treasury of the Italian Ministry of Economy and Finance.
