# Supplementary material for: Educational and gender disparities in work, retirement, and disability-pension expectancies: a cross-country analysis of Italy and Finland
Source: J Gerontol B Psychol Sci Soc Sci. 2026 Apr 8;81(6):gbag064. doi: 10.1093/geronb/gbag064 (PMC13180651; doi:10.1093/geronb/gbag064)
Supplement: gbag064_Supplementary_Data [file gbag064_supplementary_data.zip › JGSS suppl Moretti et al.docx]

# ***The Journals of Gerontology, Series B: Psychological Sciences and Social Sciences* Supplementary Material: Moretti et al. Educational disparities in work, retirement, and disability-pension expectancies: a cross-country analysis of Italy and Finland.**

**Supplementary Methods 1: Definition of the classification of individuals in the states**

We used hierarchical, mutually exclusive states to define the labour market status of the individuals. We classified all individuals to be unemployed/inactive, if they did not work, receive retirement or disability pension. Next, we classified people as working if they have been employed at least 30 days during the year and divided full-year employment and mid-to-low intensity, based on the number of months of they have been employed, including in the former those employed more than 350 days per year and in the latter, those employed between 30 and 350 days in the year. We classified people as being in retirement if they receive the old age pension, if they receive survivor pension (widowed or family members), and for Finland, we also include pension for farmers. Finally, we classified people as being in disability pension if they receive this benefit. Each new state thus overrides the previous state in the above-presented order: for example, an individual who is employed for a part of the year but also receives, at some point, disability and old age pension would be considered to be on disability pension. We aggregated unemployed and inactive because in Italian data, the individuals are observed primarily through contribution histories and pension receipt, making it impossible to consistently distinguish between unemployment and other forms of inactivity.

We have chosen the above-mentioned day thresholds to account for the difference between those who are employed all year and those who are not. Italy and Finland have a similar distribution of months being in employment, so that over 80% of workers (overall) are employed throughout the year, with a similar distribution among those being employed for 1–3 months and 4–11 months, ensuring comparability between the two countries.

The definition and eligibility criteria for disability pensions are similar in both countries. The disability pension is granted only to people having total and permanent disability, in a “financially needy condition” and whose work capacity is “significantly and permanently reduced”. The disability pension is converted into a (old age) retirement pension at around the age of 65 (at the time of the study) in both countries. One distinction is the income threshold, which varies slightly but may reflect differences in purchasing power between the two countries. Another difference relates to residency requirements: in Italy, applicants must be permanent residents, and non-EU citizens are required to hold a residence permit valid for at least one year. In Finland, applicants must have resided in the country for at least three years after the age of 16, except in cases where the individual became disabled before the age of 19 or previously received a disability allowance.

**Supplementary Methods 2: Analysis**

In the estimation of transition probabilities, the probability of transition from state $i$ to state $j$ in a one-year time interval is defined as:

$$p_{\mathrm{ij}}={P(X}_{t+1}=j | X_{t}=i)$$

and it is computed using multinomial logistic regression.

Specifically, we estimate the probability of being in state $j$ at time $t+1$, as a function of the state $i$ at time $t$ and other covariates $x$. Formally:

$$P(y_{t+1}=j | y_{t}=i, x)= \frac{exp(\alpha+ \beta_{t}y_{t-1}+\beta^{T}x)}{1+ \sum exp(\alpha+ \beta_{t}y_{t}+\beta^{T}x)}$$

where $y_{t+1}$ is the state at time $t+1$ and $y_{t}$ the state at time $t$; $i$ and $j$ represent the possible states (work full-year; work mid to low intensity; unemployed, inactive, or work less than 30 days in the year; retired; in disability pension, dead) and $x$ the covariates included in the model (namely individual's age, squared and cubic age, and education). The models are stratified by gender.

For the computation of the expectancies, starting from the transition probabilities, we composed them in a transition matrix $(U)$ and computed the fundamental matrix $(N)$ as $N={(I-U)}^{-1}$, where $I$ is an identity matrix. From $N$all quantities of interest are derived, such as the expectancies.

The expectancies computed are period-based measures, meaning that the results must be interpreted as what would happen to the synthetic cohort of individuals if the age-specific transition probabilities remained unchanged for the lifetime of the cohorts.

**Table S1**. State expectancies at age 30 (and % over total life expectancy) between 2005 and 2018 for Finnish and Italian men and women by level of education

|  | **Finland** | | | | | | | | | | | |
| --- | --- | --- | --- | --- | --- | --- | --- | --- | --- | --- | --- | --- |
|  | **men** | | | | | | **women** | | | | | |
|  | **low** | | **mid** | | **high** | | **low** | | **mid** | | **high** | |
|  |  | % |  | % |  | % |  | % |  | % |  | % |
| full | 17.2 | 39 | 21.9 | 46 | 26.9 | 52 | 16.0 | 31 | 21.5 | 40 | 26.3 | 47 |
| midlow | 3.7 | 8 | 3.5 | 7 | 2.2 | 4 | 4.6 | 9 | 4.4 | 8 | 3.1 | 6 |
| unimp | 4.1 | 9 | 2.7 | 6 | 1.8 | 3 | 6.1 | 12 | 3.1 | 6 | 2.0 | 3 |
| retir | 14.1 | 32 | 16.5 | 34 | 19.9 | 38 | 16.7 | 32 | 21.5 | 40 | 23.0 | 41 |
| disab | 5.3 | 12 | 3.4 | 7 | 1.4 | 3 | 5.8 | 11 | 3.8 | 7 | 1.7 | 3 |
| total | 44.5 |  | 47.9 |  | 52.2 |  | 52.2 |  | 54.2 |  | 56.0 |  |
|  |  |  |  |  |  |  |  |  |  |  |  |  |
|  | **Italy** | | | | | | | | | | | |
|  | **men** | | | | | | **women** | | | | | |
|  | **low** | | **mid** | | **high** | | **low** | | **mid** | | **high** | |
|  |  | % |  | % |  | % |  | % |  | % |  | % |
| full | 18.3 | 36 | 22.9 | 43 | 24.1 | 46 | 12.0 | 21 | 18.4 | 32 | 21.6 | 38 |
| midlow | 3.0 | 6 | 2.2 | 4 | 1.6 | 3 | 2.4 | 4 | 2.4 | 4 | 2.3 | 4 |
| unimp | 9.3 | 18 | 9.1 | 17 | 7.5 | 14 | 21.9 | 39 | 17.7 | 31 | 11.8 | 21 |
| retir | 14.0 | 28 | 14.7 | 28 | 16.8 | 32 | 14.2 | 25 | 14.6 | 26 | 17.5 | 31 |
| disab | 5.9 | 12 | 4.2 | 8 | 2.9 | 5 | 6.0 | 11 | 4.1 | 7 | 3.6 | 6 |
| total | 50.5 |  | 53.0 |  | 52.9 |  | 56.5 |  | 57.2 |  | 56.8 |  |

**Figure S1**. State expectancies at age 30 (%) over total life expectancy between 2005 and 2018 for Finnish and Italian men and women, by level of education
